# Peptidergic Modulation of the Lobster Cardiac System Has Opposing Action on Neurons and Muscles

**DOI:** 10.1093/iob/obaf002

**Published:** 2025-01-24

**Authors:** I S Petropoulos, A E Jordan, P S Dickinson, D J Powell

**Affiliations:** Neuroscience Program, Bowdoin College, Brunswick, ME 04011, USA; Neuroscience Program, Bowdoin College, Brunswick, ME 04011, USA; Neuroscience Program, Bowdoin College, Brunswick, ME 04011, USA; Neuroscience Program, Bowdoin College, Brunswick, ME 04011, USA

## Abstract

Modulation of neuronal networks, primarily through neuropeptides, generates variations in motor patterns that allow organisms to adapt to environmental changes or sensory inputs. Modulation is complex, with receptors for neuromodulators expressed at various locations within a nervous system; neuromodulators can thus alter muscle dynamics peripherally via the neuromuscular junction (NMJ) and the muscle itself. The neurogenic cardiac neuromuscular system of the American lobster (*Homarus americanus*) is a well-characterized model for investigating peptidergic modulation. Myosuppressin (pQDLDHVFLRFamide) is an endogenous peptide that interestingly decreases contraction frequency while also increasing contraction force by acting at both the lobster heart central pattern generator (CPG; the cardiac ganglion) and the periphery (cardiac muscles). Myosuppressin decreases heartbeat frequency by decreasing the burst frequency of the cardiac ganglion. Here, we investigated the remaining question, does myosuppressin exert its peripheral effects directly on the cardiac muscle, the NMJ, or both? To elucidate myosuppressin's effects on the cardiac muscle, the muscle was isolated from the CPG, and contractions were evoked using focal application of the endogenous neurotransmitter, l-glutamate, while superfusing myosuppressin over the heart. Myosuppressin increased glutamate-evoked contraction amplitude in the isolated muscle, suggesting that myosuppressin exerts its peripheral effects directly on the cardiac muscle. To examine effects on the NMJ, excitatory junction potentials were evoked by stimulating the motor nerve and recording the intracellular membrane voltage from a single muscle fiber both in control saline and in the presence of myosuppressin. Myosuppressin did not modulate the amplitude of excitatory junction potentials suggesting that myosuppressin acts directly on the muscle and not via the NMJ, to cause an increase in contraction force.

## Introduction

All nervous systems are modulated by molecules such as amines, amino acids, and peptides. Because receptors for these modulators can be expressed at multiple loci within a nervous system (including functionally distinct portions of the same cell, e.g., dendritic arbor vs synaptic terminal) and because receptors for the same modulator can drive distinct physiological responses, it can be inherently difficult to predict how a modulator will influence a circuit. This is even true for well-characterized circuits with known synaptic connections. Here, we use the established American lobster (*Homarus americanus*) cardiac neuromuscular system to study how modulation at more than one location occurs, and to increase our understanding of how modulation at multiple sites leads to greater dynamics than modulation at a single receptor site. Because neuropeptides act on multiple targets simultaneously, elucidating mechanisms through which they initiate, sustain, and modulate behaviors is challenging (Dickinson and Powell 2023).

Whereas the myogenic mammalian heart can contract independently of neural input, the lobster heart is neurogenic: a central pattern generating (CPG) neuronal circuit excites the cardiac muscle fibers, thus driving rhythmic contractions. The circuit responsible for rhythmic neural firing is called the cardiac ganglion (CG) (Cooke 2002; Williams et al. 2013). The CG is a Y-shaped ganglion located in the dorsal wall of the heart; it consists of nine neurons: five anterior motor neurons and four posterior premotor neurons that are chemically and electrically coupled (i.e., connected by electrotonic synapses) to generate synchronous, rhythmic, and monophasic bursting activity (Cooke 2002; Williams et al. 2013). Glutamate is the endogenous excitatory neurotransmitter in the heart, and its release by motor neurons quickly activates sodium currents (Jan and Jan 1976; Wu and Cooper 2012; Titlow and Cooper 2018). The *H. americanus* cardiac muscle consists of striated myocardial cells (striated muscle) that are nonpropagating fibers and show graded responses to neural input (Hooper and Weaver 2000; Millar et al. 2005; Sherman and Burrage 2015). Studying individual components of the system is especially tractable as the neurons, neuromuscular junction (NMJ), and muscles from the same preparation can all be studied.

The crustacean NMJ—the chemical synapse between the motor neuron and the muscle fiber—is conducive for studying the properties of synaptic transmission and integration/facilitation as both muscle contractions and excitatory junction potentials (EJPs) can be readily measured (Miller et al. 1985; Worden et al. 1997; Worden 1998; Titlow and Cooper 2018). When sufficiently depolarized, most vertebrate skeletal muscles fire action potentials, which in turn result in contractions. Similarly, for crustacean muscle contraction, neurotransmitter release at the NMJ results in a depolarization of the muscle membrane potential (i.e., the EJP); however, muscle contraction is a graded function of depolarization and thus, muscle contraction is proportional in size to the amount of depolarization (Orkand 1962; Atwood et al. 1965; Titlow and Cooper 2018). The extent to which the cardiac muscles contract as a function of EJP integration is known as the neuromuscular transform (Brezina et al. 2000a, 2000b; Brezina and Weiss 2000; Williams et al. 2013). Furthermore, while vertebrate motor neurons terminate on skeletal muscles with a large bouton called the “end plate,” invertebrate muscles are multiply innervated along the muscle fiber (Sherman and Atwood 1972; Atwood 1976).

The decapod crustacean cardiac neuromuscular system has been shown to have the capacity to be modulated at multiple sites, including the neurons, muscles, and NMJ, by a multitude of modulators including myosuppressin, C-type allatostatin (C-AST), crustacean cardioactive peptide, FMRFamide-like peptides, and proctolin (Miller and Sullivan 1981; Wilkens et al. 2005; Fort et al. 2007a, 2007b; Stevens et al. 2009; Wiwatpanit et al. 2012). One of the modulators known to act at multiple sites is the peptide myosuppressin (pQDLDHVFLRFamide) (Stevens et al. 2009). Myosuppressin perfused through the whole heart causes a rapid decrease in contraction frequency and force followed by a large increase in force (contraction amplitude). In the isolated CG, myosuppressin increases the duration of action potential bursts and decreases their cycle frequency by hyperpolarizing motor neuron membrane potentials. Thus, the decrease in the contraction frequency can largely be attributed to myosuppressin's effects on the CG; however, these changes in neuronal output do not fully explain the changes in contraction force. Stevens et al. (2009) showed that when a terminal segment of nerve (i.e., still connected to muscles but with the CG itself removed) was stimulated to drive muscle contractions, myosuppressin increased the amplitude of cardiac contractions relative to control. This suggests that, in addition to its effects on the CG, myosuppressin modulates either the NMJ, the muscle, or both sites directly.

To determine whether the myosuppressin-induced increase in cardiac muscle contraction force is due to myosuppressin's effect on the NMJ, the muscle itself, or both, we conducted two types of experiments: (1) we used focally applied glutamate to evoke muscle contractions in the absence of the CG (dissected from the heart), thus bypassing the NMJ such that we could independently study responses of the transverse muscles to myosuppressin, and (2) we stimulated a segment of motor nerve in order to generate single EJPs such that we could examine whether or not myosuppressin affected EJP dynamics independently from muscle contractions. Because there are a variety of processes, both pre- and postsynaptic, that can be modulated and affect attributes of an EJP, we quantified EJP amplitude, decay, and facilitation.

## Methods

### Animals

Adult (∼500 g) *H. americanus* were purchased from local seafood retailers in Brunswick, ME, USA. Individuals were housed in recirculating natural seawater aquaria and were maintained at 10–12°C on a 12-h/12-h light/dark cycle. Female and male animals at various molt stages were used. They were fed once a week, with a diet of chopped shrimp or squid. Experimental and animal care procedures were performed following protocols approved by Bowdoin College.

### Experimental setup and lobster dissection

In preparation for physiological experiments, individual lobsters were anesthetized by packing in ice for ∼30 min prior to dissection. The heart is positioned anterior to the tail, attached to the dorsal carapace. Hearts were dissected from the body of the animal; for recordings of both glutamate-induced contractions and EJPs, the heart was then further dissected from the cephalothoracic carapace (with the hypodermis attached). After dissection, preparations were maintained in chilled (8–10°C) physiological lobster saline (composition in mM: 479.12 NaCl, 12.74 KCl, 13.67 CaCl_2_, 20.00 MgSO_4_, 3.91 Na_2_SO_4_, 11.45 Trizma base, and 4.82 maleic acid); if need be, pH was adjusted to 7.45 using NaOH or HCl (pH measured at room temperature), as described in Dickinson et al. (2018). The heart was opened along the ventral axis to expose the intact CG and cardiac muscles under a dissecting microscope. The subsequent dissection for each preparation is described below.

Physiological saline was superfused across the heart with a Rabbit peristaltic pump (Gilson, Middleton, WI, USA) at a flow rate of ∼5 mL/min for the isolated muscle and EJP preparations. Throughout all experiments, the temperature of the physiological saline was maintained between 10 and 12°C via a Peltier temperature regulator (CL-100 bipolar temperature controller and SC-20 solution heater/cooler; Warner Instruments, Hamden, CT, USA) and was monitored with a temperature probe (Warner Instruments, Hamden, CT, USA).

### Myosuppressin synthesis and preparation

Myosuppressin (pQDLDHVFLRFamide; MW = 1272.46 g/mol) was custom synthesized by GenScript Corporation (Piscataway, NJ, USA) and bath applied using the peristaltic pump. Myosuppressin has relatively low aqueous solubility and was dissolved in dimethyl sulfoxide (DMSO, 15%) and then diluted in deionized water to make 10^−3^ M stock solutions (Stevens et al. 2009). The 10^−3^ M myosuppressin stock solution was stored in small aliquots at −20°C and diluted in room temperature lobster saline to desired concentrations (10^−6^ or 10^−7^ M) immediately before experimental use.

### *In vivo* heart recordings

Lobster hearts were dissected while remaining attached to the overlaying section of the dorsal thoracic carapace to preserve the natural stretch existing in the intact animal. The posterior artery of the heart was cannulated via a short piece of tubing and continuously perfused with physiological saline at a flow rate of 2.5 mL/min. To record heart contractions, the anterior arteries were tied off (with 6/0 suture silk) at a 30–45° angle from the horizontal plane to a Grass FT03 force-displacement transducer (Astro-Med, West Warwick, RI, USA). The contraction output was amplified using an ETH-250 Bridge amplifier (CB Sciences, Dover, NH, USA) with a high-pass filter (4 Hz), and further amplified using a Brownlee 410 amplifier (Brownlee Precision, San Jose, CA, USA).

### Glutamate-evoked cardiac muscle contractions

**Fig. 1:**
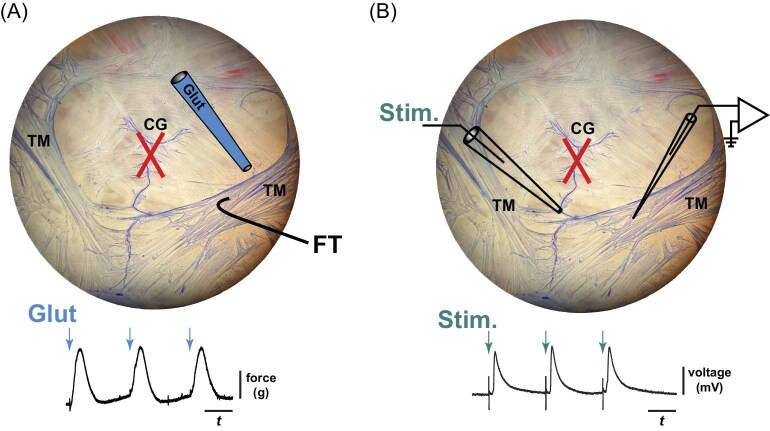
Physiological approaches. The modulatory effects of myosuppressin on cardiac muscle were assessed in two ways: (**A**) measuring changes in glutamate-evoked muscle contractions, and (**B**) measuring changes in the postsynaptic responses (excitatory junction potentials; EJPs) that were evoked by stimulating the terminal segment of the posterior lateral motor nerve that innervates the transverse muscle (TM). (**A**) Photograph of the dissected lobster heart that has been cut along the ventral, rostral–caudal axis to reveal the cardiac ganglion (CG) and associated cardiac muscles. The preparation was stained using methylene blue to visualize nerve and muscle tissue. A force transducer (FT) was used to measure contractions in one of the TM. Contractions were evoked in the absence of the CG (dissected out; indicated by the “X” over the CG) via focal application of glutamate (Glut, 5.5 × 10^−4^ M). A representative force transducer trace is shown below the photograph with blue arrows indicating three instances of focally applied glutamate. (**B**) The same photograph used in **(A)** to show a schematic of how postsynaptic excitatory junction potentials (EJPs) were recorded. Similar to **(A)**, the CG was dissected away from the heart; however, in this approach, a segment of terminal nerve (no neurons present) was left intact so that a suction electrode (Stim.) could be used to stimulate the motor nerve. In this way, we were able to record single EJPs from the TM fibers using a sharp electrode (schematic on the right). EJPs were then amplified and recorded; an example recording is shown below the photograph with teal arrows indicating when each nerve stimulus was delivered (voltage transients are visible on the voltage trace).

To ask whether myosuppressin exerts its effects directly on the muscle itself, cardiac muscle contractions were measured using either of the two lateral, transverse muscles (Fig. 1A) located in the interior of the lobster heart. The CG was removed to eliminate spontaneous neural input (spontaneous contractions). Removing the CG both eliminated intrinsic neuronal stimulation of the muscle fibers and bypassed the NMJ, allowing us to independently assess muscle activity in response to myosuppressin. After a healthy bundle of muscle fibers was located, hooks were glued perpendicular to each end of the bundle using GluTure topical tissue adhesive (Zoetis Inc., Kalamazoo, MI, USA). Healthy bundles were identified by visualizing contractions of the muscle fibers driven by the CG (prior to CG removal). Before glue was applied, the fiber bundle was briefly dried to remove any physiological saline from the region, ensuring the glue would adequately dry. During this process, the preparation was out of saline for less than 30 s, which caused no known harm to the heart (Qu 2017). The optical force transducer was placed on top of the muscle between the hooks. After isolating the cardiac muscle, muscle contractions were stimulated via focal application of l-glutamate, the endogenous neurotransmitter released by the CG motor neurons. l-Glutamate was administered via an Aladdin Single-Syringe pump (WPI, Sarasota, FL, USA) that was connected to a glass pipette (OD: 1 mm × ID: 0.75 mm, 4 in. in length; Cat# 615000, AM Systems, Sequim, WA, USA) positioned to focally apply glutamate (5.5 × 10^−4^ M) onto one of the lateral muscles with the following parameters: 0.02 mL was applied every 250 s at a rate of 0.85 μL/min (i.e., as a 1.41-s application). Concentration was empirically determined via a set of experiments observing sensitization/desensitization with glutamate concentrations ranging from 10^−3^–10^−4^ M. Physiological saline was constantly perfused across the heart until the glutamate-evoked muscle contractions were stable in size. Once contractions exhibited a consistent amplitude, we bath applied myosuppressin (10^−6^ M) over the heart for 30 min while periodically applying glutamate (0.85 μL/min, 0.02 mL every 250 s with a 1.41-s application). Following the 30-min myosuppressin application, the preparation was washed with physiological saline to ensure that glutamate-evoked contractions returned to baseline (same amplitude/width as control) [Results].

Muscle contractions were measured using a SI-H optical force transducer (WPI Inc., Sarasota, FL, USA) and amplified via a model 1700 A-M Systems Differential AC Amplifier (Sequim, WA, USA) (methods adapted from Maguire 2019). Voltage signals were digitized and converted to a measure of contraction force using a Micro 1401 digitizer (CED, Milton, Cambridge, UK). Signals were recorded using Spike2 software (CED, Milton, Cambridge, UK) on a Dell PC (Dell, Austin, TX, USA). We only analyzed data from preparations in which the contractions returned to baseline—indicating healthy and undamaged heart muscle (*N* = 10/11).

### Recording EJPs

To stimulate and record EJPs, the heart was opened as described earlier, and contracting transverse muscle fibers were identified visually. In some cases, those fibers were impaled in this semi-intact heart to record spontaneous bursts of EJPs. For experiments involving myosuppressin, however, the CG (including all neuronal somata) was removed to eliminate EJPs driven by spontaneous neuronal activity. To record EJPs, muscle fibers were then impaled with a sharp microelectrode, pulled using a Sutter P-97 (Sutter Instruments, Novato, CA, USA), containing a solution that mimics the intracellular solution in the muscle (squid cytoplasmic fill; Hooper et al. 2015). Electrode resistances ranged from 12 to 22 MΩ. Resting membrane potentials of recorded muscle fibers ranged from −30 to −70 mV. Voltage signals were amplified using an Axoclamp-2B (Axon Instruments/Molecular Devices, San Jose, CA, USA), digitized using a Micro 1401 (CED, Milton, Cambridge, UK) and recorded using Spike2 software (CED, Milton, Cambridge, UK) on a Dell PC (Dell, Austin, TX, USA).

For this preparation, a terminal segment of the posterior lateral nerve was left intact so that axonal fibers could be stimulated via a suction electrode. Glass suction electrodes pulled on a DMZ-Universal Puller (Zeitz-Instrumente Vertriebs GmbH, Planegg, Germany) were hand cut and fire polished using a microforge (MF-830 Narishige, Tokyo, Japan) to have a 100-μm opening at the tip. A model 2100 isolated pulse stimulator (A-M Systems, Sequim, WA, USA) or a Grass S88 dual output square pulse stimulator (Grass Instruments) was used to stimulate the cut end of the motor nerve. Stimuli (0.5-ms duration) were delivered in trains of three or four at a frequency of 2.5 Hz within a train; trains were generated at 0.01 Hz. A range of voltages (2–7 V) was used as each nerve/preparation required a different threshold voltage to evoke an EJP. Once EJPs (Fig. 1B) were elicited consistently, myosuppressin was superfused over the preparation for 20 min (Stevens et al. 2009). For these experiments, myosuppressin was used at 10^−7^ M to decrease muscle movement to ensure stable recordings. Changes in membrane potential in response to stimulation or myosuppressin were recorded using an intracellular electrode.

## Data analysis

### The cardiac muscle

Glutamate-evoked contractions were analyzed using a custom script in Spike2 to measure the peak of the contraction and the baseline tension. To compare the amplitude and baseline changes of the contractions in physiological saline and in myosuppressin, the four contractions elicited prior to myosuppressin application in each preparation were compared to four contractions in the myosuppressin at peak response (within subject; *N* = 10). Decay time constants were calculated using a custom script in MATLAB as the amount of time it took the muscle to relax to ∼63% (1 − e^−1^) from its peak contraction in response to focal glutamate application.

### The neuromuscular junction

The amplitudes of the stimulated EJPs (EJP 1, EJP 2, and EJP 3) were manually measured using the “peak finder” function and the horizontal cursors function in Spike2. Due to facilitation, the EJPs within each train were analyzed individually as three separate groups, EJP 1, 2, and 3, in the control saline, myosuppressin, and the wash. In some cases, four EJPs were evoked. For these, we only analyzed the first three EJPs such that we could compare them with experiments where only three EJPs were stimulated. Seven EJP trains were analyzed in each condition (control and myosuppressin) for each preparation. To quantify facilitation, we used a facilitation index, which was the amplitude of the third EJP divided by the first.

### Statistical analysis of cardiac muscle and NMJ experiments

Statistical analysis was carried out using both MATLAB (MathWorks Inc., Natick, MA, USA) and Graphpad Prism (Dotmatics, Woburn, MA, USA). Prism software was also used for making all plots. Because data were not normally distributed, Wilcoxon sign-rank tests were used for within-subject comparisons between saline and myosuppressin conditions (*α* = 0.05). Because nonparametric tests were used to compare groups, all descriptive statistics are reported as median ± standard deviation (SD).

## Results

### Myosuppressin increased the amplitude of glutamate-evoked contractions in the isolated cardiac muscle

**Fig. 2:**
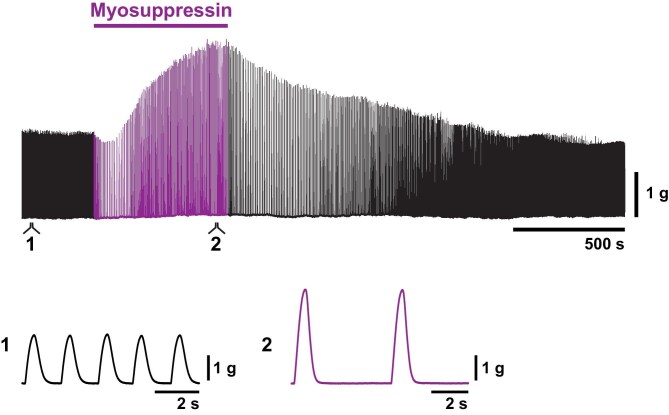
Myosuppressin elicited a complex response in the whole lobster heart. This representative recording shows a compressed trace from a force transducer affixed to the whole heart during perfusion of myosuppressin (10^−6^ M; bar over recording/purple section of the trace). Expanded portions of the recording (1) and (2) are shown below the compressed trace to clearly show both the decrease in heartbeat frequency and the increase in contraction force that occur in the presence of myosuppressin compared to the control condition (perfusion of physiological saline). Each contraction is driven by a burst of action potentials from the CG motor neurons.

Myosuppressin (10^−6^ M) perfusion through the whole heart results in a decrease in contraction frequency and a biphasic modulation (small decrease then larger increase) of contraction amplitude (Stevens et al. 2009) (Fig. 2, purple segment of the force transducer recording). Myosuppressin receptor mRNA is expressed in the cardiac muscle tissue as well as the CG (Oleisky et al. 2020, 2022), suggesting that these receptor proteins are expressed in both tissues and therefore may uniquely modulate each. This may, in part, explain the unusual response observed when perfusing myosuppressin through the whole heart. In particular, the effect of myosuppressin on muscle receptors may result in the increased contraction amplitude observed in the whole heart recordings (Oleisky et al. 2020, 2022).

**Fig. 3:**
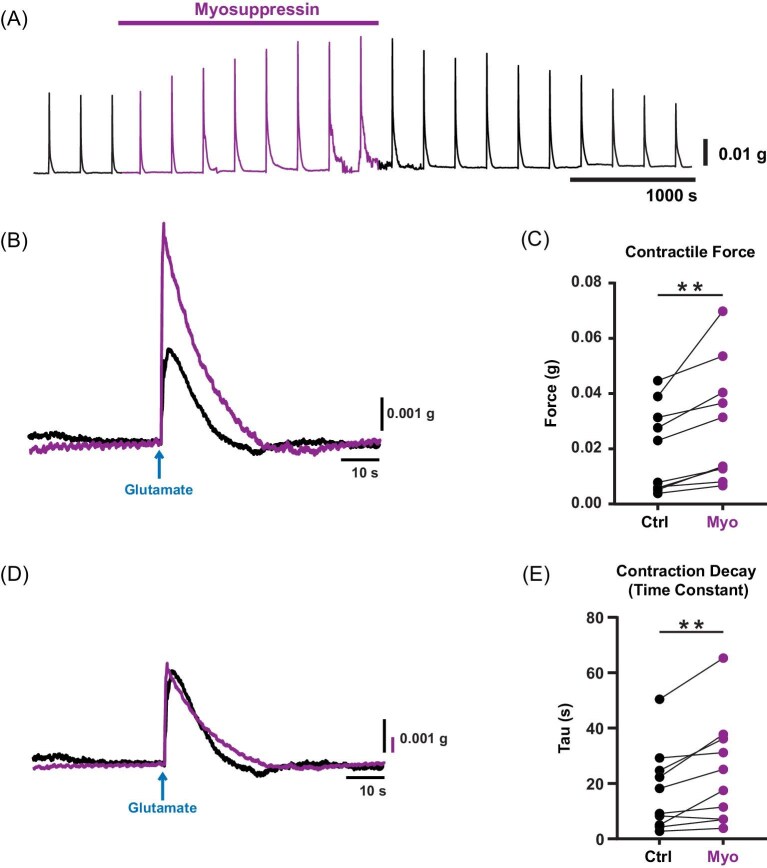
Myosuppressin increased glutamate-evoked contractile force as well as relaxation time in the transverse muscles. (**A**) A representative trace showing the effects of myosuppressin (10^−6^ M; application duration indicated by the bar over the physiological trace and the purple portion of the trace) on the contraction force of a single transverse muscle. Each contraction was elicited via focal application of glutamate (5.5 × 10^−4^ M). During the time course of the myosuppressin application, glutamate-evoked contractions increased in amplitude. (**B**) Two glutamate-evoked contractions from the same preparation in control (black) and myosuppressin (purple) overlayed and time-locked to the glutamate application, showing the relative increase in contraction force. (**C**) Plot of contraction force in control (ctrl, black) and myosuppressin (myo, purple). Each point represents the mean responses from a single preparation, and black lines link the same preparation in control and myosuppressin. We observed an increase in contraction force in the presence of myosuppressin (Wilcoxon sign-rank test: ctrl: 0.015 ± 0.015 g, myo: 0.023 ± 0.021 g; *P* = 0.002, *N* = 10). (**D**) The same two glutamate-evoked contractions shown in (**B**) overlayed and normalized to the same peak force, illustrating the increase in relaxation time. (**E**) Plot of relaxation time constant in control (ctrl, black) and myosuppressin (myo, purple). Each point represents the mean responses from a single preparation, and black lines link the same preparation in control and myosuppressin. We observed an increase in the relaxation time constant in the presence of myosuppressin (Wilcoxon sign-rank test: ctrl: 13.7 ± 14.9 s, myo: 21.3 ± 19.1 s; *P* = 0.006, *N* = 10). Values are median ± SD.

To determine whether the muscle responses to myosuppressin were taking place at the level of muscle rather than the NMJ, we stimulated muscle contractions independently of the NMJ by activating contractions with focally applied l-glutamate, the endogenous excitatory neurotransmitter in the heart. To control for any mechanical disturbance as a result of expelling solution from a micropipette, we focally applied lobster saline onto the transverse muscle. We did not observe any response from the force transducer during these saline applications (data not shown, *N* = 5). Once consistent contractions were established, myosuppressin (10^−6^ M) was superfused over the cardiac muscle, and muscle contractions in myosuppressin were compared to those recorded in saline alone. Myosuppressin increased glutamate-evoked contraction force in the isolated transverse muscles (Fig. 3A–C; Wilcoxon sign-rank test, control [ctrl]: 0.015 ± 0.015 g, myosuppressin [myo]: 0.023 ± 0.021 g, *P* = 0.002, *N* = 10), demonstrating that myosuppressin acts directly on the cardiac muscle. Additionally, myosuppressin increased the relative rate of muscle relaxation as measured by the relaxation time constant (Fig. 3D and E; Wilcoxon sign-rank test, ctrl: 13.7 ± 14.9 s, myo: 21.3 ± 19.1 s, *P* = 0.006, *N* = 10). Specifically, the decay of contraction increased in the myosuppressin application (Fig. 3D and E). Although myosuppressin generally increased the time constant of relaxation, it is important to note that contraction duration was highly variable and inconsistent across preparations.

**Fig. 4:**
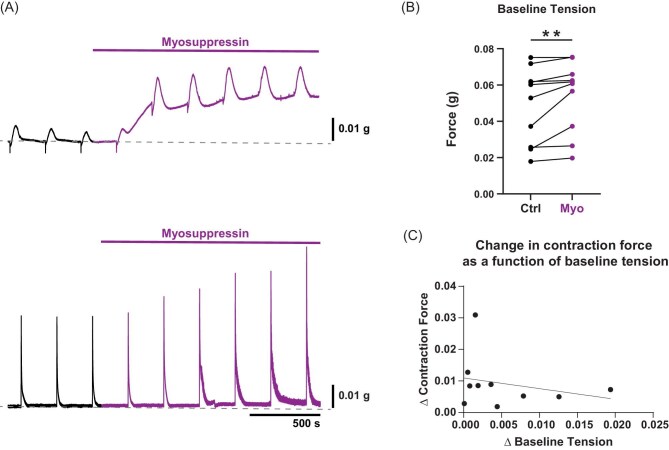
Myosuppressin increased muscle tone. (**A**) Two force transducer recordings (different preparations) demonstrating that, in the presence of myosuppressin, the transverse muscle resting tension (gray dashed line) increased. The upper trace shows the greatest response observed in resting tension, whereas the resting tension in the preparation shown in the lower trace increased only slightly. Even in the preparation showing minimal increases in resting tension, the amplitude of glutamate-evoked contractions increased considerably, suggesting that the increase in muscle tone is not responsible for the increased amplitude of evoked contractions. (**B**) Plot of resting tension for both control and myosuppressin conditions, myosuppressin (Wilcoxon sign-rank test: ctrl: 0.057 ± 0.021 g, myo: 0.061 ± 0.020 g; *P* = 0.002, *N* = 10). Values are median ± SD. (**C**) Plot of the change in glutamate-evoked contraction force due to myosuppressin as a function of the change in baseline tension due to myosuppressin application (Spearman's test *ρ* = −0.31, *P* = 0.39, *N* = 10). Data points are matched pairs between each metric, and the solid line represents the best-fit line from a linear model (*r*^2^ = 0.07, *P* = 0.47).

Myosuppressin perfusion through the whole heart tends to produce a fairly stereotyped response (Fig. 2) that is not accompanied by a change in the resting tension of the heart as measured from the anterior arteries [Methods]. Interestingly, we observed a subtle, myosuppressin-induced increase in muscle baseline tension when measuring the contraction force of the transverse muscles, albeit with a large degree of variation between preparations (Fig. 4A). In some cases, resting muscle tension increased substantially relative to the amplitude of the contraction response to focally applied glutamate (Fig. 4A, *upper trace*), while in other cases, resting muscle tension changed very little in myosuppressin (Fig. 4A, *lower trace*). Although we did observe an increase in muscle baseline tension across preparations (Fig. 4B; Wilcoxon Sign-Rank test, ctrl: 0.057 ± 0.021 g, myo: 0.061 ± 0.020 g, *P* = 0.002, *N* = 10), we did not observe a correlation between the change in baseline tension and the change in glutamate-evoked peak contraction force between saline and myosuppressin conditions (Fig. 4C; Spearman's test, *ρ* = −0.31, *P* = 0.39, *N* = 10). Thus, while contraction force can be influenced by resting muscle tension (Dickinson et al. 2016), we did not observe a correlation between these two changes in the cardiac muscle physiology in response to myosuppressin. This finding contrasts with what we observe in the whole heart recording, where we do not observe a change in resting muscle tension when measuring heartbeat force along the rostral–caudal axis (control: 2.08 ± 0.5 g, myosuppressin: 2.07 ± 0.4 g; Wilcoxon sign-rank test: *P* = 0.76, *N* = 14; data not shown).

### Myosuppressin does not have a significant effect on stimulated EJPs

**Fig. 5:**
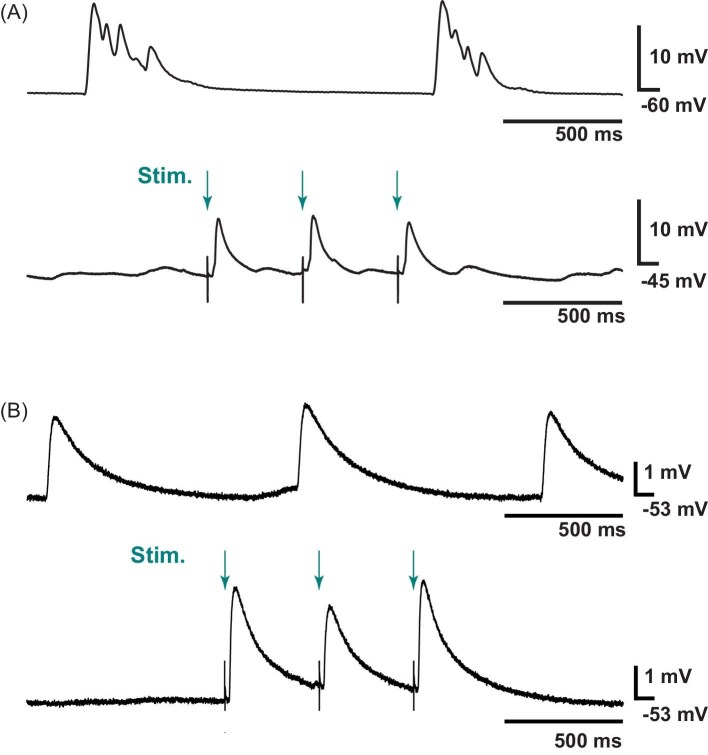
Variation in spontaneous and evoked EJPs. (**A**) Top trace shows an example recording of the EJPs recorded from an intact preparation (CG not dissected out) where spontaneous EJPs occurred in bursts and had voltage deflections of ∼15 mV. Lower trace shows that for some stimulated muscle preparations, we observed EJPs that were similar in amplitude to spontaneously observed EJPs, in this case ∼10mV. Each trace is from a different preparation. (**B**) In some cases, spontaneous EJPs were smaller in amplitude (upper trace, ∼3 mV); however, in the same preparation we observed a similar voltage deflection when EJPs were stimulated (lower trace, ∼3.5 mV). Arrows and stimulus artifacts indicate the time of stimulation.

Crustacean muscle contraction is a graded function of depolarization, with many muscles being multiply innervated. In crustaceans, muscle action potentials are not generated, and the amplitude of muscle contraction is proportional to the extent of EJP-driven depolarization. EJPs typically occur in bursts driven by the bursts generated by the CG (Fig 5A, *upper trace*). However, even when the CG was present, we recorded individual EJPs in some preparations (Fig 5b, *upper trace*). In addition to the patterning of these EJPs, these recordings illustrate the variability in EJP amplitude between muscle fibers. To enable us to examine the effects of myosuppressin superfusion on EJP amplitude, EJPs were stimulated at a sufficiently slow frequency to minimize facilitation, ensuring stable recordings through the experiment and allowing us to observe individual EJPs clearly (Fig. 5A and B, *lower traces in each panel*).

**Fig. 6:**
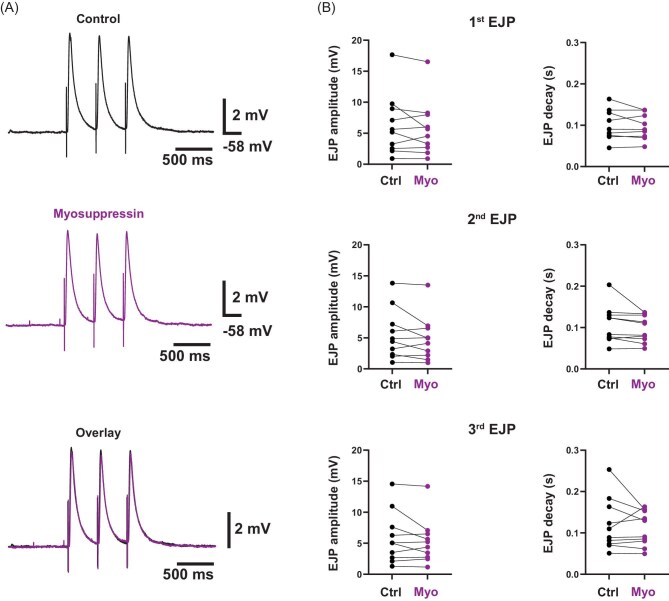
Myosuppressin does not affect EJPs. (**A**) Representative traces from the same preparation showing that EJPs stimulated in control conditions (top, black) and myosuppressin (middle, purple) do not differ in peak voltage or decay. Lower trace is an overlay to show the similarity in EJPs between the two conditions. EJPs were elicited in groups of three (triplicates) at a frequency of 2.5 Hz. (**B**) Plots comparing the first, second, and third EJPs in each stimulated triplicate. Myosuppressin did not affect the EJP voltage deflections (Wilcoxon sign-rank tests: EJP 1: ctrl: 5.4 ± 4.9 mV, myo: 5.1 ± 4.5 mV; *P* = 0.49, *N* = 10; EJP 2: ctrl: 4.6 ± 4.1 mV, myo: 4.5 ± 3.7 mV; *P* = 0.32, *N* = 10; EJP 3: ctrl: 5.0 ± 4.2 mV, myo: 4.8 ± 3.6 mV; *P* = 0.51, *N* = 10) or decay time constants (Wilcoxon sign-rank tests: EJP 1: ctrl: 0.10 ± 0.04 s, myo: 0.10 ± 0.37 s; *P* = 0.74, *N* = 10; EJP 2: ctrl: 0.10 ± 0.05 s, myo: 0.10 ± 0.03 s; *P* = 0.055, *N* = 10; EJP 3: ctrl: 0.01 ± 0.06 s, myo: 0.11 ± 0.04 s; *P* = 0.77, *N* = 10). Values are median ± SD.

Because the pattern of neuronal and muscular expression of the myosuppressin receptors is unknown (and there is no working antibody for these receptors), it is possible that these receptors are expressed at the NMJ. If so, myosuppressin could either augment presynaptic release of glutamate or modulate the postsynaptic response to glutamate, or both concurrently. Therefore, myosuppressin binding presynaptically at the NMJ could contribute to the increased contraction amplitude observed in the whole heart recordings. If myosuppressin were modulating the NMJ, we would expect to observe a myosuppressin-induced increase in EJP amplitude. Thus, we stimulated trains of action potentials in the motor nerve to generate trains of EJPs that we recorded in the muscle fibers in saline (control) and myosuppressin (10^−7^ M). Figure 6A shows the EJP responses to a train of three stimuli in both saline (top trace) and myosuppressin (middle trace) from a single preparation. The lower trace in Fig. 6A shows an overlay of both traces to demonstrate the similarity in EJPs elicited in each condition. We did not observe any changes in EJP amplitude or voltage decay, suggesting that myosuppressin does not modulate EJP dynamics (Fig. 6B; EJP amplitude—Wilcoxon sign-rank tests: EJP 1: ctrl: 5.4 ± 4.9 mV, myo: 5.1 ± 4.5 mV; *P* = 0.49, *N* = 10; EJP 2: ctrl: 4.6 ± 4.1 mV, myo: 4.5 ± 3.7 mV; *P* = 0.32, *N* = 10; EJP 3: ctrl: 5.0 ± 4.2 mV, myo: 4.8 ± 3.6 mV; *P* = 0.51, *N* = 10; EJP decay time constants—Wilcoxon sign-rank tests: EJP 1: ctrl: 0.10 ± 0.04 s, myo: 0.10 ± 0.37 s; *P* = 0.74, *N* = 10; EJP 2: ctrl: 0.10 ± 0.05 s, myo: 0.10 ± 0.03 s; *P* = 0.055, *N* = 10; EJP 3: ctrl: 0.01 ± 0.06 s, myo: 0.11 ± 0.04 s; *P* = 0.77, *N* = 10). Each of the three EJPs in a train was analyzed individually (EJP 1, EJP 2, and EJP 3) to account for possible effects of facilitation, although we did not observe any facilitation at the frequency of EJPs we used here; moreover, myosuppressin did not lead to facilitation (facilitation index [median ± SD], control: 1.03 ± 0.2 vs myosuppressin 10^−7^ M: 1.03 ± 0.2; Wilcoxon sign-rank test, *P* = 0.63, *N* = 10 [data not shown]).

Thus, while we observed an increase in muscle contraction force and baseline tension in the cardiac transverse muscles of the lobster heart in the presence of myosuppressin, the modulatory peptide did not alter EJP physiology. Therefore, our data support the hypothesis that the observed response in the lobster heart to myosuppressin (Fig. 1) is due to modulation of the CG neurons (Stevens et al. 2009) and the cardiac muscles, but not due to modulation of the NMJ.

## Discussion

### Myosuppressin exerts effects at the periphery

Peptidergic neuromodulation is dynamic and depends on various factors, including modulator concentration, responses on different timescales (e.g., ion channel modulation vs changes in transcription), and the tissue-specific receptor expression patterns. In the latter case, receptors can be expressed at physiologically distinct compartments in the same cell and/or expressed in two adjacent cells such that hormonally released modulators will impact circuits globally. In neuronal circuits where neurons have reciprocal synaptic connections or in neuromuscular circuits where muscle contractions feedback to modulate neuron activity, the impact of hormonally released modulators would be impossible to predict without a deep mechanistic understanding of each component (Dickinson and Powell 2023). Even in well-defined invertebrate circuits where neuron connections are known, circuit responses to modulatory signaling molecules result in nonlinear responses with a large pool of possible outcomes that are difficult to predict (Jorge-Rivera and Marder 1996; Jorge-Rivera et al. 1998; Swensen and Marder 2000, 2001; Fort et al. 2007a, 2007b).

Here, we demonstrate that by applying glutamate (the neuromodulator released by the CG motor neurons) directly on the muscle, we could measure the modulation of the excitation–contraction coupling (measured here as contraction amplitude) in this system in the absence of the NMJ. This was important because NMJ, and therefore EJP, modulation can be mechanistically complex. EJP attributes (i.e., amplitude, facilitation, decay, etc.) are controlled by a multitude of pre- and postsynaptic processes. Briefly, the fact that we did not observe any difference between the EJPs evoked in saline and those evoked in myosuppressin indicates that myosuppressin does not modulate any of the processes that govern the presynaptic release of neurotransmitter, nor does it regulate the receptor channels that ultimately lead to an EJP by allowing the flow of ionic current. Thus, we can confidently conclude that the action of myosuppressin on the cardiac neuromuscular system occurs through two distinct tissues and that the physiological response in each tissue is not influenced by the other (i.e., we do not have to consider changes in the neuromuscular transform that would then affect excitation–contraction coupling).

Here, we show that a peptide modulator, known to decrease the burst frequency of the CG (Stevens et al. 2009), simultaneously increases the cardiac contraction force via direct action on the cardiac muscles (Fig. 1). Not only is this dissimilar to the effects of other peptide modulators that enhance muscle contraction dynamics (detailed below), but it is also unique when compared to other modulators that decrease CG burst frequency. For instance, the C-type allatostatin peptides (AST-Cs) often decrease the lobster CG burst frequency, but this is accompanied by a decrease in contraction force (Wiwatpanit et al 2012). In some cases, AST-C application increases CG burst frequency; however, in these cases, an increase in contraction force was often also observed (Dickinson et al. 2018; Muscato et al. 2022). Williams et al. (2013) explored the neuromuscular transform in this system and demonstrated that AST-C-driven changes in contraction force are largely attributed to/driven by changes in frequency and duty cycle. Interestingly, the decreases in contraction frequency and simultaneous increases in contraction force in response to myosuppressin are not explained by the neuromuscular transform. This may in part be due to the fact that the EJPs are not modulated by myosuppressin. Thus, one interesting feature of modulating the neurons and muscles independently, but not the EJPs, is that the output of the neuromuscular system is not constrained by the neuromuscular transform. While the reason for dual and opposing regulation of these neurons and muscle fibers is not immediately clear, this exemplifies the complex nature of modulation despite the relatively low dimensionality of this preparation (nine neurons, muscle fibers, and the associated synaptic processes).

The absence of EJP modulation is unique compared to peptide modulators that increase neuron excitability and enhance muscle contraction force, as these peptides also modulate EJP amplitude (Jorge-Rivera and Marder 1996; Jorge-Rivera et al. 1998; Wilkens et al. 2005). EJPs stimulated in the gastric mill muscles 4 and 6 (gm4, gm6) of the crab, *Cancer borealis*, were shown to increase in amplitude in response to TNRNFLRFamide, serotonin, proctolin, and dopamine, and slightly decrease in amplitude in response to C-AST. Although the changes in EJP amplitude were smaller than the observed contraction force, EJP amplitude did change, following the same directional trend in all modulators. For example, nerve-evoked contractions and EJPs increased in amplitude in response to TNRNFLRFamide; however, the change in EJP amplitude (<20%) was modest compared to the 120% increase in contraction force (Jorge-Rivera and Marder 1996).

At least three of the same peptides shown to modulate gastric mill muscle contraction (Jorge-Rivera and Marder 1996,^ 1997^; Jorge-Rivera et al. 1998) are known to modulate the cardiac neuromuscular system: TNRNFLRFamide, proctolin, and AST-C (Miller et al. 1985; Wilkens et al. 2005; Wiwatpanit et al 2012). However, the only peptide for which effects on EJPs have been extensively studied in the lobster heart is proctolin (Miller and Sullivan 1981; Wilkens et al. 2005). Indeed, proctolin increases EJP amplitude in a manner similar to what is observed in the gastric mill, suggesting many parallels between the systems. Thus, while it has not been shown that other peptides alter EJP amplitude in the lobster cardiac neuromuscular system, it is nevertheless surprising that myosuppressin did not alter EJP amplitude as a part of its modulatory action on the cardiac muscles. However, studies of other crustacean neuromuscular systems have shown that aspects of the neuromuscular system other than EJPs are likewise subject to peripheral neuromodulation. For example, in the lobster stomatogastric system, dopamine has been shown to evoke spiking in isolated peripheral axons, including most of the peripheral axon of the pyloric dilator neurons (Bucher et al. 2003; Daur et al. 2019). Additionally, TNRNFLRFamide modulation of nerve-evoked contractions in muscles near the anterior cardiac plexus has been shown to differ qualitatively across three crab species, suggesting that FLRFamides (like myosuppressin) may have evolved to play distinct roles in modulating stomatogastric muscle movements in different *Cancer* species (Verley et al. 2008).

In addition to increasing contraction amplitude, myosuppressin increases the relaxation time constant. Due to the presence of stretch feedback in the lobster heart (muscle stretch negative-feedback results in a slowing of neuron burst generation), this slowing of muscle relaxation could help to maintain a decreased, stable heartbeat frequency. In *Aplysia*, two members of the small cardioactive peptide family (SCPA and SCPB) and serotonin increase the amplitude and relaxation rate of the accessory radula closer muscle contractions (Weiss et al. 1978, 1992). It is hypothesized that it is important for the rate of relaxation to increase when contraction amplitude increases, so that these muscles, which are an important part of the *Aplysia* feeding apparatus, are able to fully relax during each feeding cycle (Weiss et al. 1978, 1992; Fox and Lloyd 1997; Lum et al. 2005). The parallel changes in contraction amplitude and relaxation rate may perform a similar function in the lobster heart, although the decrease in contraction frequency that is elicited by myosuppressin's effect on the CG neurons may decrease the importance of changes in relaxation time constant relative to those in *Aplysia*.

In some cases, myosuppressin changed the baseline tension in the transverse muscle, likely due to properties specific to the muscle itself, as this phenomenon is not observed in whole heart preparations. As such, it is possible that myosuppressin only changes transverse muscle tone and does not affect longitudinal muscle baseline tension. Indeed, previous work provides evidence for differing muscle force between dimensions in response stretch (Dickinson et al. 2016). However, it is also possible that the lack of observed changes in baseline tension could be due to the imperfect way in which whole heart contractions were sampled; the force transducer cannot be aligned with the rostral–caudal axis of the heart, which would otherwise produce high-fidelity measurements of contraction force and, thus, we may have missed subtle changes in tension.

### Myosuppressin could exert different effects through a range of signaling pathways

The precise mechanism underlying the myosuppressin-induced decrease in CG bursting and increase in muscle contraction has not been identified. Although the myosuppressin peptide terminates with FLRFamide motif, it has been shown in the fruit fly, *Drosophila melanogaster*, to have a distinct receptor, and therefore not be a functional member of the FLRFamide-like-peptide (FLP) family (Dickerson et al. 2012). Therefore, even though many of the other peptides in the FLP family have been previously studied in crustacean nervous systems, it is possible that the myosuppressin receptor expressed in the cardiac neuromuscular system differs substantially from other FLP receptors. One hypothesis is that there is a distinct expression of different myosuppressin receptors in each tissue (neural and muscular) that leads to the observed differing responses. Oleisky et al. (2020) provided evidence that myosuppressin, when applied to the motor neurons and pacemaker interneurons separately, produced unique responses. Furthermore, they observed distinct receptor mRNA transcripts in each CG cell type (premotor vs motor neurons).

In a follow-up study, Oleisky et al. (2022) showed that the nonamidated myosuppressin (pQDLDHVFLRFG) only modulated the nervous system but did not affect the muscle. This indicates that receptors present in the muscle could be different (and/or driving distinct intercellular signaling pathways) than those in the CG specifically, suggesting that there is at least one receptor that acts at the CG and not at the muscle. Interestingly, all five known myosuppressin receptors are seen to be present in the muscle (Oleisky et al. 2022). However, it is possible that one of the receptors in the muscle could be expressed at such low levels that it is not contributing to the muscle response. Alternatively, because we applied saturating concentrations of myosuppressin (10^−6^ M), we could be observing an occlusion effect with some high-affinity receptors generating a low-concentration-specific effect, which is occluded by low-affinity receptor responses activated by this high concentration of myosuppressin. This later hypothesis has been demonstrated for dopamine in the lobster stomatogastric nervous system (Rodgers et al. 2011; Krenz et al. 2013; Rodgers et al. 2013; Krenz et al. 2015).

### Proctolin, which modulates the muscle through L-type calcium channels, provides a model for a mechanism for myosuppressin's effects on the cardiac muscle

Several mechanisms could explain the myosuppressin-induced increase in contraction amplitude. Given that EJPs remain unchanged with myosuppressin, the receptors are likely extrasynaptic. Although we have not yet addressed further mechanistic possibilities, proctolin provides a unique case study that may parallel myosuppressin's effects on the muscle. Proctolin modulates both NMJ and muscle physiology in the lobster heart (Wilkens et al. 2005). Wilkens et al. (2005) showed that the extent of the lobster's cardiac muscle contraction was proportional to calcium increase, suggesting that proctolin affects calcium dynamics. Mechanistically, proctolin was shown to affect calcium release from the l-type calcium channels in the sarcoplasmic reticulum (SR). When an l-type calcium blocker (ryanodine) was applied, contractions were suppressed even when proctolin was administered, indicating that sequestered calcium (from the SR) is essential to cause enhanced contractions. Caffeine elicits release from the SR and when applied in the presence of proctolin, the observed contractions were faster and stronger (compared to contractions with no caffeine) (Wilkens et al. 2005). Understanding the calcium dynamics in conjunction with myosuppressin would be an interesting next step in this work; it is very possible that myosuppressin could be acting in the same manner as proctolin, by inducing increased release of calcium from internal stores.

## Conclusions

Taken together, these data suggest that myosuppressin acts on the isolated cardiac muscle, but does not modulate EJPs, to drive an increase in contraction amplitude in the lobster heart. Myosuppressin provides a unique example of a modulator in which the global effects of modulation are well-characterized. This includes appreciating myosuppressin's modulation on distinct CG neurons (Oleisky et al. 2020, 2022) as well as the muscle fibers.

## Supplementary Material

obaf002_Supplemental_File

## Data Availability

Raw data and metadata are available on Zenodo: 10.5281/zenodo.14706945
