# Supplementary material for: Peptidergic Modulation of the Lobster Cardiac System Has Opposing Action on Neurons and Muscles
Source: Integr Org Biol. 2025 Jan 24;7(1):obaf002. doi: 10.1093/iob/obaf002 (PMC11816307; doi:10.1093/iob/obaf002)
Supplement: obaf002_Supplemental_File [file obaf002_supplemental_file.docx]

**Figure 1) Physiological approaches.** The modulatory effects of myosuppressin on cardiac muscle were assessed in two ways: **A)** measuring changes in glutamate-evoked muscle contractions, and **B)** Measuring changes in the post-synaptic responses (excitatory junction potentials; EJPs) that were evoked by stimulating the terminal segment of the posterior lateral motor nerve that innervates the transverse muscle (TM). **A)** Photograph of dissected lobster heart that has been cut along the ventral, rostral-caudal axis to reveal the cardiac ganglion (CG) and associated cardiac muscles. The preparation was stained using methylene blue to visualize nerve and muscle tissue. A force transducer (FT) was used to measure contractions in one of the Transverse Muscles (TM). Contractions were evoked in the absence of the CG (dissected out; indicated by the red X over the CG) via focal application of glutamate (Glut, 5.5 x 10^-4^ M). A representative force transducer trace is shown below the photograph with blue arrows indicating three instances of focally applied glutamate. **B)** The same photograph used in **(A)** to show a schematic of how postsynaptic excitatory junction potentials (EJPs) were recorded. Similar to **(A)**, the CG was dissected away from the heart; however, in this approach, a segment of terminal nerve (no neurons present) was left intact so that a suction electrode (Stim.) could be used to stimulate the motor nerve. In this way, we were able to record single EJPs from the transverse muscle (TM) fibers using a sharp electrode (schematic on the right). EJPs were then amplified and recorded; an example recording is shown below the photograph with teal arrows indicating when each nerve stimulus was delivered (voltage transients are visible on the voltage trace).

**Figure 2) Myosuppressin elicited a complex response in the whole lobster heart.** This representative recording shows a compressed trace from a force transducer affixed to the whole heart during perfusion of myosuppressin (10^-6^ M; purple bar over recording/purple section of the trace). Expanded portions of the recording (1) and (2) are shown below the compressed trace to clearly show both the decrease in heartbeat frequency as well as the increase in contraction force that occur in the presence of myosuppressin compared to the control condition (perfusion of physiological saline).

**Figure 3) Myosuppressin increased glutamate-evoked contractile force as well as relaxation time in the transverse muscles. A)** A representative trace showing the effects of myosuppressin (10^-6^ M; purple portion of the trace) on the contraction force of a single transverse muscle. Each contraction was elicited via focal application of glutamate (5.5 x 10^-4^ M). During the time course of the myosuppressin application, glutamate-evoked contractions increased in amplitude. **B)** Two glutamate-evoked contractions from the same preparation in control (black) and myosuppressin (purple) overlayed and time-locked to the glutamate application, showing the relative increase in contraction force. **C)** Plot of contraction force in control (ctrl, black) and myosuppressin (myo, purple). Each point represents the mean responses from a single preparation, and black lines link the same preparation in control and myosuppressin. We observed an increase in contraction force in the presence of myosuppressin (Wilcoxon Sign-Rank test: ctrl: 0.015±0.015 g, myo: 0.023±0.021 g; *p* = 0.002, *N* = 10). **D)** The same two glutamate-evoked contractions shown in **(B)** overlayed and normalized to the same peak force, illustrating the increase in relaxation time. **E)** Plot of relaxation time constant in control (ctrl, black) and myosuppressin (myo, purple). Each point represents the mean responses from a single preparation, and black lines link the same preparation in control and myosuppressin. We observed an increase in the relaxation time constant in the presence of myosuppressin (Wilcoxon Sign-Rank test: ctrl: 13.71±14.9 s, myo: 21.30±19.1 s; *p* = 0.006, *N* = 10). Values are median±SD.

**Figure 4) Myosuppressin increased muscle tone.** **A)** Two force transducer recordings (different preparations) demonstrating that, in the presence of myosuppressin, the transverse muscle resting tension (gray dashed line) increased. The upper trace shows the greatest response observed in resting tension, whereas the resting tension in the preparation shown in the lower trace increased only slightly. Even in the preparation showing minimal increases in resting tension, the amplitude of glutamate-evoked contractions increased considerably, suggesting that the increase in muscle tone is not responsible for the increased amplitude of evoked contractions. B) Plot of resting tension for both control and myosuppressin conditions, myosuppressin (Wilcoxon Sign-Rank test: ctrl: 0.057±0.021 g, myo: 0.061±0.020 g; *p* = 0.002, *N* = 10). Values are median±SD. C) Plot of the change in glutamate-evoked contraction force due to myosuppressin as a function of the change in baseline tension due to myosuppressin application (Spearman’s test *ρ* = -0.31, *p* = 0.39, *N* = 10). Data points are matched pairs between each metric, and the solid line represents the best-fit line from a linear model (*r^2^* = 0.07, *p* = 0.47).

**Figure 5) Variation in spontaneous and evoked EJPs. A)** Top Trace: shows an example recording of the EJPs recorded from an intact preparation (CG not dissected out) where spontaneous EJPs occurred in bursts and had voltage deflections of ~15mV. Lower trace: shows that for some stimulated muscle preparations, we observed EJPs that were similar in amplitude to spontaneously observed EJPs, in this case ~10mV. Each trace is from a different preparation. **B)** In some cases, spontaneous EJPs were smaller in amplitude (upper trace, ~3mV), however, in the same preparation we observed a similar voltage deflection when EJPs were stimulated (lower trace, ~3.5mV). Arrows and stimulus artifacts indicate the time of stimulation.

**Figure 6) Myosuppressin does not affect EJPs. A)** Representative traces from the same preparation showing that EJPs stimulated in control conditions (top, black) and myosuppressin (middle, purple) do not differ in peak voltage or decay. Lower trace is an overlay to show the similarity in EJPs between the two conditions. EJPs were elicited in groups of three (triplicates) at a frequency of 2.5 Hz. **B)** Plots comparing the first, second, and third EJP in each stimulated triplicate. Myosuppressin did not affect the EJP voltage deflections (Wilcoxon Sign-Rank tests: EJP 1: ctrl: 5.40±4.90 mV, myo: 5.12±4.51 mV; *p* = 0.49, *N* = 10; EJP 2: ctrl: 4.64±4.05 mV, myo: 4.54±3.65 mV; *p* = 0.32, *N* = 10; EJP 3: ctrl: 5.03±4.17 mV, myo: 4.81±3.64 mV; *p* = 0.51, *N* = 10) or decay time constants (Wilcoxon Sign-Rank tests: EJP 1: ctrl: 0.101±0.037 s, myo: 0.097±0.0.32 s; *p* = 0.74, *N* = 10; EJP 2: ctrl: 0.103±0.045 s, myo: 0.096±0.032 s; *p* = 0.055, *N* = 10; EJP 3: ctrl: 0.010±0.063 s, myo: 0.110±0.042 s; *p* = 0.77, *N* = 10). Values are median±SD.
